# Household Storage of Medicines and Associated Factors in Tigray Region, Northern Ethiopia

**DOI:** 10.1371/journal.pone.0135650

**Published:** 2015-08-14

**Authors:** Abrham Wondimu, Fantahun Molla, Birhanu Demeke, Tadele Eticha, Admassu Assen, Solomon Abrha, Wondim Melkam

**Affiliations:** Department of Pharmacy, College of Health Sciences, Mekelle University, Mekelle, Ethiopia; Nottingham University, UNITED KINGDOM

## Abstract

**Introduction:**

The presence of medicines in households is a risk factor for irrational drug use. This study aimed at investigating the prevalence and factors associated with home storage of medicines in Tigray Region, Ethiopia.

**Method:**

A community based cross-sectional study was conducted in April 2013 in Tigray Region, Ethiopia. A total of 1034 participants were enrolled in the study. A multi-stage sampling method was employed to select households. Data were collected with the help of a pre-tested structured questionnaire and analyzed using descriptive statistics and bivariate and multivariate logistic regression.

**Result:**

Of the total households visited, 293(29%) stored drugs. The mean number of drugs per household was 1.73. The most common classes of drugs found in households were analgesics 149(29%) and antibiotics 128(25%). Most of the medicines kept in households were used for ongoing treatments 316(62%) and available in tablet dosage form (70%). More than half of the medications kept at homes were not adequately labeled while drawer 180(36%) were reported as the main place of drug storage. The proportion of home storage of medicines in rural area (AOR = 0.56, 95% CI: 0.39–0.81) was lower than that of urban area. However, households having family member(s) working in health facilities (AOR = 2.03, 95% CI: 1.09–3.77) were associated with an increased home storage of medicines.

**Conclusion:**

Most drugs kept at home were not appropriately labeled and stored in a safe place. Residence area (rural versus urban) and the presence of health professional(s) in the households affects household drug storage. Hence, public education campaign should be considered as an intervention to improve the storage condition of medicines in the households.

## Introduction

Pharmaceutical research has steadily brought new drugs to the market, aimed at cure or palliation of numerous diseases. Their wide accessibility provides important benefits in reducing morbidity and mortality and alleviating pain and suffering [1]. However, improved availability, if combined with poor compliance by patients and inappropriate self-medication, may result in wastage of resources, increased resistance of pathogens, serious health hazards, adverse reactions, and, indeed, prolonged suffering [2, 3].

Worldwide in most households, medicines are kept for various purposes including emergency use and treatment of chronic or acute illnesses. Drug storage at home is a risk factor in relation to irrational drug use mainly due to the easy access, and improper storage. If the recommendations for storage are not followed, the drug stability can be affected which in turn leads to ineffective drug therapy [4, 5]. On the other hand, controlling the use of drugs stored at home is a great task especially from unintentional users such as children which increases the risk of accidental poisoning. Moreover, presence of medicines at home has also been associated with sharing of drugs which further increase the risk of inappropriate drug use and hence the emergence of antimicrobial resistance [4].

Many studies in Africa identified a high prevalence of drug storage at home. In Sudan, about 98% of investigated families had at least one drug product stored at home [6]. Study conducted in Uganda also showed that about 40% of the surveyed households kept medicines at home and 30% of identified antibacterials found in surveyed households were kept for future use [7]. In Ethiopia, a study conducted almost two decades ago in Addis Ababa revealed that 20% of the studied households were found hoarding drugs, and drug sharing was practiced by 17% of the respondents [8]. Apart from this study, little has been done to characterize drugs stored in households in Ethiopia. Therefore, this study aimed at generating data on the prevalence and factors associated with home storage of medicines in Tigray Region, Northern Ethiopia.

## Methods and Materials

### Study Area and Design

A community based cross-sectional study was conducted in April 2013 in Tigray Region, Northern Ethiopia. Tigray regional state consists of seven administrative zones: Western, Northwestern, Central, Eastern, Southeastern and Southern, and one special zone which is Mekelle. The capital city of the region is Mekelle which is located 783 kilometers north of Addis Ababa, the capital city of Ethiopia. Based on the 2007 Census conducted by the Central Statistical Agency (CSA) of Ethiopia, the Region has an estimated total population of 4,314,456 and 985,654 households with averagely 4.4 persons per household [9]. In each household, data were collected from the household head or any adult household member (≥18 years) present at home during the time of data collection.

### Sample size determination and sampling procedure

To determine the sample size a formula for two population proportions based on the prevalence of medicine users in urban area 74% [8] and rural area 61.5% [10] was used. Using β = 0.2, 5% margin of error at 95% confidence level, and considering 5% non-response rate and design effect of 2 the calculated sample size was 1034.

A multi-stage sampling method was employed to select the sample. In stage I, taking the homogeneity of population of seven zones into consideration, two Zones (Southern Tigray and Eastern Tigray Zones) were selected from the Tigray Regional state by simple random sampling technique. In stage II, one rural district and one urban administrative town (urban district) were selected from each of the selected Zones at random. In stage III, households were recruited for the study from the selected urban and rural areas by systematic random sampling and proportional to size allocation technique.

### Data collection and analysis

Data on the presence of medicines in households and their utilization were collected trained pharmacists using a structured questionnaire (S1 File). To ensure quality of the data, the questionnaire was pretested in 50 households in similar setups before the actual data collection. Data on socio-demographic characteristics, sources of drugs, and reasons for stocking drugs at home were collected through interview and data about drug storage condition, dosage form, expiry date and situation of packaging material were collected by direct observation from each households included in the study.

Data were coded, checked for completeness and consistency. Data were entered using Epi-Data version 3.1 and analyzed using SPSS version 16.0 statistical software. Bivariate logistic regression analysis was performed between socio-demographic factors related to medications kept at home to identify risk factors related to home storage of drugs. Factors significantly associated with home storage of drugs in the bivariate analysis were included in a multivariate logistic regression model. All p values were two tailed with the significance level set at 0.05.

### Ethical Considerations

The study was approved by the Health Research Ethics Review Committee of College of Health Sciences, Mekelle University. After approval for ethical issues, official letters of cooperation were obtained from Tigray Regional Health Bureau and given to the respective community leaders. The purpose of the study was explained to the study population, confidentiality was ensured and written consent was obtained before data collection.

## Results

### Socio-demographic characteristics

Overall, 1000 (97%) households responded to the interview, among them 504 urban and 496 were rural. The median family size of the households was 5 with just above half (52%) of the households had at least five family members. More than half (55%) of the households had no children less than five years while 34% had one child and the rest had at least two children. Two in five households had fathers with below primary education while about half (49%) of the households had illiterate mothers. Only 7% of the surveyed households had health professional(s) as a family member. Distribution of socio-demographic characteristics of the households is shown in Table 1.

**Table 1:** Socio-demographic characteristics of households based on the prevalence of home storage of medicines in Tigray.

Characteristics		Home storage of medicines		COR (95% CI)	p value	AOR (95% CI)	p value
		Yes, n (%)	No, n (%)				
Residence	Urban	187 (37)	317 (63)	1		1	
	Rural	106 (21)	390 (79)	0.46 (0.35, 0.61)	0.0	0.56 (0.39, 0.81)	0.0
Family size	<5	136 (28)	348 (72)	1			
	≥5	157 (30)	359 (70)	1.12 (0.85, 1.47)	0.4		
# of children < 5 years	0	162 (30)	388 (71)	1			
	1	100 (30)	237 (70)	1.01 (0.75, 1.36)	0.9		
	≥2	31 (27)	82 (73)	0.91 (0.58, 1.42)	0.7		
# of people > 65 years	0	212 (29)	530 (71)	1			
	≥1	81 (31)	177 (69)	1.14 (0.84, 1.56)	0.4		
Father’s education level	Illiterate	34 (23)	145 (77)	1		1	
	Read & write	58 (27)	161 (74)	1.22 (0.77, 1.91)	0.4	0.97 (0.60, 1.57)	0.9
	Primary education	36 (29)	89 (71)	1.36 (0.82, 2.28)	0.2	0.91 (0.50, 1.66)	0.8
	Secondary education	34 (34)	67 (66)	1.71 (1.00, 0.92)	0.0	1.04 (0.54, 2.00)	0.9
	Tertiary education	47 (45)	58 (55)	2.73 (1.64, 4.57)	0.0	1.59 (0.76, 3.31)	0.2
Mother’s education level	Illiterate	114 (23)	376 (77)	1		1	
	Read & write	50 (30)	115 (70)	1.43 (0.97, 2.12)	0.1	1.18 (0.72, 1.92)	0.5
	Primary education	44 (34)	85 (66)	1.71 (1.12, 2.60)	0.0	1.40 (0.79, 2.49)	0.3
	Secondary education	40 (40)	60 (60)	2.20 (1.40, 3.45)	0.0	1.55 (0.83, 2.89)	0.2
	Tertiary education	37 (43)	50 (58)	2.44 (1.52, 3.92)	0.0	0.94 (0.44,2.03)	0.9
Presence of health professional in the family	No	262 (28)	671 (72)	1		1	
	Yes	31 (46)	36 (54)	2.21 (1.34, 3.64)	0.0	2.03 (1.09, 3.77)	0.0

### Prevalence of home storage of medicines

Of the total households visited, 293 stored drugs. Higher proportion of households in urban (19%) stocked medicines at home as compared to that of the rural area (11%). The mean number of drugs found per household was 1.73.

Categories of medicines kept in households and their utilization are indicated in Table 2. The most common classes of drugs found in the households were analgesics (29%) and antibiotics (25%). Generally, more than half (62%) of the medications were used for ongoing treatment.

**Table 2:** Categories of medicines found in households.

Category	Status of medicines at home			
	Current use	Leftover	Anticipated future use	Total
Analgesics	59 (12)	53 (10)	37 (7)	149 (29)
Antibiotics	70 (14)	54 (11)	4 (1)	128 (25)
Antihypertensive drugs	49 (10)	-	-	49 (10)
ART	39 (8)	-	-	39 (8)
Antacids	22 (4)	14 (3)	2 (0)	38 (8)
Antiasthmatics	11 (2)	5 (1)	4 (1)	20 (4)
Minerals & vitamins	8 (2)	9 (2)	1 (0)	18 (4)
Antidiabetics	9 (2)	1 (0)	-	10 (2)
Hormonal drugs/ contraceptives	10 (2)	-	-	10 (2)
Antihelmentics	9 (2)	-	-	9 (2)
Antiepileptics	4 (1)	-	-	4 (1)
Others	26 (5)	5 (1)	2 (0)	33 (7)
Total (%)	316 (62)	141 (28)	50 (10)	507 (100)

**Fig 1:**
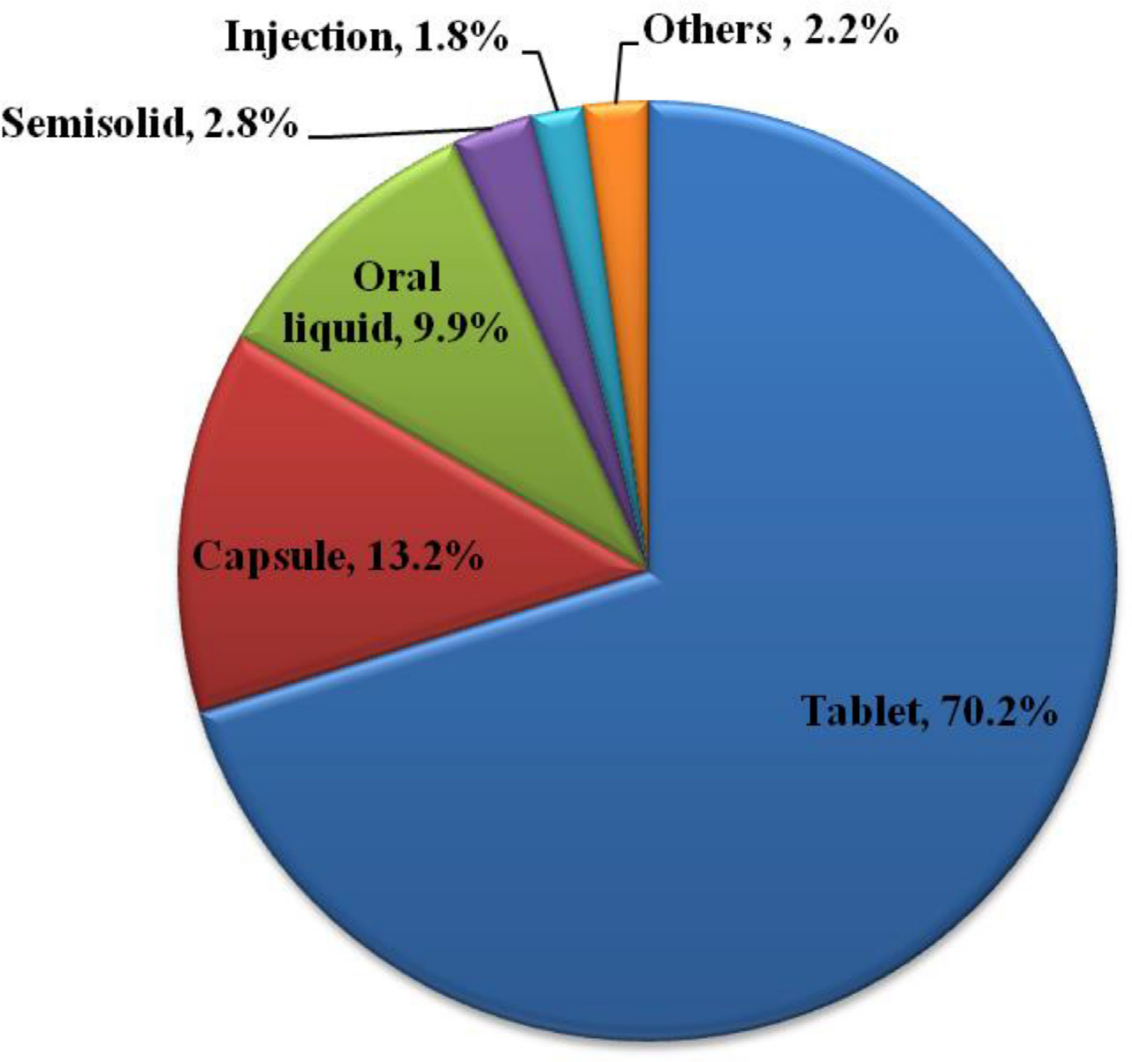
Dosage form of medicines found in the household. It indicates routes of administration of medications kept at home in Tigray region, northern Ethiopia.

Dosage form of medicines kept in households is shown in Fig 1, most (70%) of the medicines were available in the form of tablets. Source of medicines and their labeling status, situation of primary package, situation of expiry date and storage place of medications kept at home are indicated in Table 3.

**Table 3:** Factors related to medications kept at home.

Description	Frequency	Percentage
**Source of medicines**		
Hospital	176	35
Health center	162	32
Pharmacy or drug store	140	28
Self, family or friend	13	3
Others	16	3
**Labeling**		
Adequately labeled	209	41
Not Adequately labeled	298	59
**Situation of primary package**		
Primary package OK	446	88
Primary package not OK	61	12
**Situation of expiry date**		
Not expired	442	87
Expired	23	5
Not known	42	8
**Storage place**		
Drawer	180	36
Cupboard	177	35
Table/Shelf	100	20
Bag	30	6
Refrigerator	20	4

### Multivariable logistic regression of the predictors of home storage of drugs

Results of logistic regression analysis are shown in Table 1. Although educational status of the fathers and mothers of the households was significantly related to home storage of drugs in the bivariate analysis, they were retained in the multivariate model as confounders of residence and presence of health professionals in the family. Results from the multivariate logistic regression analysis indicated that the residence of the people was significantly associated with home storage of drugs. Households found in urban area had high chance of storing drugs in comparison to rural area households (AOR = 0.56, 95% CI: 0.39–0.81). On the other hand, households with family member(s) working in the health facilities (AOR = 2.03, 95% CI: 1.09–3.77) were more likely to store medicines at home.

## Discussion

Taking drugs at home without prescription has become a habit that is often encouraged in the community [6]. The result of this study revealed that 29% of the visited households stored drugs. This value seems to be more or less similar to the findings of the previous studies done in Ethiopia [8, 11] and it is less prevalent as compared to the studies done elsewhere [4, 5, 9]. The lower prevalence of home drug storage in Ethiopia could be attributed to the fact that substantial number of Ethiopians are relying on traditional medicine [12, 13].

The majority of medicines present at homes were mostly for ongoing treatments (62%) in this study. This is in agreement with other studies done in Uganda [4] and Iraq [14]. In agreement with other studies [14, 15, 16], significant proportion of drugs (28%) found in the households were left over from the previous illness in this study. People may keep leftover drugs because of initial excessive prescribing for treatment, inadequate adherence to treatment and anticipated future use [14]. For instance, in the present study, 10% of the medicines were stocked in the home anticipating future need. The same reason was also reported in different studies [2, 6, 14]. Frequent drug stock outs and inaccessibility of adequate health care in developing countries like Ethiopia might be the possible explanation [4,17].

The mean number of drugs per household was 1.73 in this study. Other studies [3,4,6,14] reported a higher number of drugs in each household. Storage of large quantities of medications at home could lead to medication administration error, accidental poisoning, adverse drug reactions, and waste of resources [3]. Residence of the people and presence health professional as a family member were significantly associated with home storage of drugs in the current study. People living in rural areas were less likely to store drugs at home. Less coverage of modern health care facilities in developing countries and dependency on traditional medicine could explain the lower home drug storage in rural area [13]. On the other hand, high proportion of home drug storage in families with health professional as a household member might be due to improvement of health seeking behavior which in turn leads the households to take drugs and control their health.

Analgesic and antibiotics were the most frequently stored categories of the drug at home in this study similar to other surveys [4, 18, 19]. Particularly, the storage of antibiotics was reported in significant number of studies [6, 14] including the present study. This has to be considered seriously because uncontrolled and excessive antibiotic use resulted from home drug storage could lead to antimicrobial drug resistance [20]. Therefore, appropriate educational campaign has to be in place to raise the awareness of the society on the appropriate utilization of antibiotics, the avoidance of leftover antibiotics, the correct disposal of leftover drugs, and harmful effects of consuming non-prescribed antibiotics [21].

Majority of home stored dosage forms were tablets followed by capsules. This was in accordance with the studies conducted in Northern Uganda [4] and Philippines [22]. High availability and ease of administration could contribute to the highest consumption rate of these dosage forms [6]. According to WHO, the medicine is considered to be adequately labeled if the label displays patient name, drug name, dose, frequency and duration of treatment [23]. Accordingly, the present study showed that majority of the drugs (59%) kept at home were not adequately labeled. This value is lower than the value (92.0%) reported in study done in Oman [17] and it is comparable with the value (59%) of the study done in Kenya [24].

In this study, majority of the drugs were stored in a drawer (36%) and cupboard (35%). This finding is in agreement with the findings reported in Palestine [15]. From this, it can be easily understood that the place and condition of storage of drugs were not appropriate and in fact the storage places were accessible to children which can lead to accidental ingestion of oral drugs by children. The expiry date of a medicine is valid if the medicine stored at the proper conditions. Around 5% of the drugs were expired in this study. Similar findings on storage of expired drugs were reported in different studies [6, 16, 19]. Lack of knowledge on expired drugs and their method of disposal could be put as possible reasons for the households to keep expired drugs [25]. Public education regarding the nature and risk of expired drugs and disposal of unused medication are needed to reduce the impact of expired drugs on the health of the community [25].

The main limitation of this study was that a cross sectional study was employed which might suffer from temporal relationship establishment with some variables and could not provide much more substantial evidence of causality, unlike a longitudinal design.

## Conclusion

The prevalence of household drug storage in Tigray region was 29%. Analgesics and antibiotics were found to be the most commonly stored drugs. Most drugs kept at home were not appropriately labeled or stored at a safe place. Residence (rural versus urban) and the presence of health professional(s) in the households were found to be associated with drug storage at home. Therefore, appropriate educational campaign has to be in place to raise the awareness of the society on appropriate utilization of drugs, avoidance of leftover drugs, correct disposal of leftover drugs and harmful effects of consuming non-prescribed antibiotics.

## Supporting Information

S1 File Questionnaire used to assess the presence of medicines in households and their utilization. (PDF)
