# Supplementary material for: Household Storage of Medicines and Associated Factors in Tigray Region, Northern Ethiopia
Source: PLoS One. 2015 Aug 14;10(8):e0135650. doi: 10.1371/journal.pone.0135650 (PMC4537258; doi:10.1371/journal.pone.0135650)
Supplement: S1 File — (PDF) [file pone.0135650.s001.pdf]

Questionnaire:

Questionnaire used to assess the presence of medicines in households and their utilization.

Part I: Socio-demographic information

1. Community:

☐ Urban ☐ Rural

2. Number of family members______________________
3. Number of family members < 5 years of age__________________
4. Number of family members > 65 years of age_________________
5. Educational level of the father:

☐ Illiterate ☐ Secondary school

☐ Read and write only ☐ College and above

☐ Primary school

6. Educational level of the mother:

☐ Illiterate ☐ Secondary school

☐ Read and write only ☐ College and above

☐ Primary school

7. Is there any member in the family working in a health sector?

☐ Yes ☐ No

8. If yes, specify his/her Job _______________________________

Part II. Information on medicines available in the households (currently used or left-over)

1. Do you have any medicines available at home today?

☐ Yes ☐ No

2. Can I please see all of them? *Write one medicine per row, and use codes provided in each column to collect information about each medicine.*

S1 Table. Information about each medicine

S/N	Medicine	Dosage form	Obtained from	In home because	Adequacy of Labeling	Primary package OK	Expiry date	Storage place
	Write name of medicine if not known, write the most detailed category given by respondent (antibiotic, antimaleria, for fever)	1=tablet<br>2=capsule<br>3=injectable<br>4=oral liquid<br>5=semisolid<br>6=others (specify)	1= family, friend<br>2=public hospital<br>3=NGO/mission hospital<br>4=public health center or dispensary<br>5=private health care provider<br>6=traditional healer<br>7=private pharmacy<br>8=supermarket/kiosk<br>9=others (specify)	1=current treatment<br>2=left from past treatment<br>3= anticipate future need	1=Yes<br>2=No<br>Yes if label include patient name, drug name dose, frequency, duration of t/t.<br>Otherwise No.	1=Yes<br>2=No<br>Yes if primary package is an envelope or a closable container and if it contains only one medicine.<br>Otherwise No.	1= not expired<br>2=expired<br>3=not known	1=refrigerator,<br>2=cupboard<br>3= drawer<br>4= other (specify)
1								
2								
3								
4								
5								
6								

The End!

The interviewer thank the respondent for giving his/her time.

መጠይቅ ቅጥዒ

ክፍሊ ሀ ፣ ማህበረ-ስነ-ህዝባዊ መረዳእታ

1. ዝነብረሉ ማሕበረሰብ

☐ ከተማ ☐ ገፀር

2. በዝሒ ስድራ ቤት __________

3. በዝሒ አባላት ስድራ ቤት ትሕቲ 5 ዓመት ዕድሚኦም ______________

4. በዝሒ አባላት ስድራ ቤት ልዕሊ 65 ዓመት ዕድሚኦም ______________

5. ደረጃ ትምህርቲ አቦ

☐ ዘይተምሃረ ☐ ካልኣይ ደረጃ ዘጠናቐቐ

☐ ምንባብን ምፅሓፍን ጥራይ ዝኽእል ☐ ኮሌጅ ዘጠናቐቐን ካብኡ ንላዕልን

☐ ቀዳማይ ደረጃ ዘጠናቐቐ

6. ደረጃ ትምህርቲ ኣዶ

☐ ዘይተምሃረት ☐ ኻልኣይ ደረጃ ዘጠናቐቐት

☐ ምንባብን ምፅሓፍን ጥራይ እትኽእል ☐ ኮሌጅ ዘጠናቐቐትን ካብኡ ንላዕልን

☐ ቀዳማይ ደረጃ ዘጠናቐቐት

7. ካብ ስድራ ቤትኩም ውሽጢ ኣብ ጥዕና መዳይ ዝሰርሕ ሰብ አሎዶ?

☐ እወ ☐ የለን

8. መልሶም/ን "እወ" እንተኾይኑ ስርሑ/ሓ'ዶ ይገልፁልና?____________________

ክፍሊ ለ፡ ብዛዕባ ኣብ ገዛ ዝረከቡ መድሓኒታት ዝገልፅ ሓበሬታ (ሐዚ ዝጥቀምሉ ወይ ዘይጥቀምሎም)

1. ኣብ ገዛኹም ኣብዚ ሐዚ እዋን መድሓኒት ኣሎዋም'ዶ?

☐ ኣሎ ☐ የለን

2. "ኣሎ" ተኾይኑ ምላሾም ክርእዮም ይፈቅዱለይ'ዶ?

ሓበሬታ፡ ቀፂሉ ዘሎ ቅጥዒ ተጠቒሞም ይመዝግብዎም፡፡ሓደ መድሓኒት ኣብ ሓንቲ መስመር ይፅሓፉ። ነቲ መድሓኒት ክምዝግቡ ከለው ሚስጥራዊ ኽም ይጠቀሙ።

ሰንጠረዥ. ንመድሓኒት ሓበሬታ

ቁ.	መድሓኒት	እቲ መድሓኒት ንተጠቃሚ ዝተዳለወ መልክዕ	እቲ መድሓኒት ዝተረኸበሉ (ዝተገዝኣሉ ቦታ)	ኣብ ገዛ ዝተቀመጠሉ ምኽንያት	ኣብቲ መድሓኒት ዝተፅሓፈ ሓበሬታ	ኹነታት እቲ መድሓኒት	መጥቀሚ ጊዜኡ ናይቲ መድሓኒት	ኣቐማ ምጣ እቲ መድሓኒት
	ሽም እቲ መድሓኒት ይፅሓፉ ተዘይተፈሊጡ በቲ ተሓታቲ ዝህቦ ሓበሬታ ምድብ እቲ መድሓኒት ይፅሓፍ	1. ኪኒን<br>2. ሽጉጥ ኪኒና<br>3. መርፍእ<br>4. ፈሳሲ(ብኣፍ ዝውሰድ)<br>5. ዝቅባእ<br>6. ኻልኦት ተሃልዩም ይዘርዝሩ	1. ካብ ቤተሰብ፣ ዓርኪ<br>2. ካብ ናይ ህዝቢ ሆስፒታል<br>3. ካብ ገበርቲ ሰናይ ሆስፒታል<br>4. ካብ ህዝቢ ጣብያ ጥዕና<br>5. ካብ ናይ ግሊ ጣብያ ጥዕና<br>6. ካብ ናይ ባህላዊ ሓኪም<br>7. ካብ ናይ ግሊ ፋርማሲ<br>8. ካብ ቤት ሹቅ<br>9. ካብ ካሊእ ተኾይኑ ይዘርዝርዎም	1. ሐዚ ዝጥቀመሉ ሰብ ስለዘሎ<br>2. ካብ ዝሓለፈ ተሓካሚ ዝተረፈ<br>3. ንዝመፅእ ሕማም ንምጥቓም	1. እኹል ሓበሬታ ኣለዎ<br>2. እኹል ሓበሬታ የብሉን<br><br>**(እኹል ሓበሬታ ክበሃል ተኾይኑ ናይቲ ሕሙም ሽም ፣ሽም እቲ ፈውሲ፣ መጠኑ፣ በዝሒ ድግግም ዝወስደሉን በዝሒ ግዘ ዝወስደሉ ዘርኢ እንተኾይኑ እዩ)**	1. ፅቡቅ<br>2. ፅቡቅ ኣይኮነን<br><br>**ፅቡቅ ክበሃል ተኾይኑ እቲ መትሓዚ (መዐሸጊ) ዝኸደን ተኾይኑን ሓደ ዓይነት መድሓኒት ተሒዙን**	1. ኣይሓለፎን<br>2. ሓሊፍዎ<br>3. ኣይፍለጥን	1. ኣብ ፍርጅ<br>2. ኣብመደርደሪ<br>3. ኣብ ተስሓቢ<br>4. ኻሊእ ተሃሊዩ ይዘርዝር
1								
2								
3								
4								
5								
6								

ተዛዚሙ

ግዜኹም ሰዊእኹም ንዚ ቃለ ማሕተት ስለዝተሓባበርኹምና ካብ ልቢ ነመስግን
